# Disentangling the Legal and Illegal Wildlife Trade–Insights from Indonesian Wildlife Market Surveys

**DOI:** 10.3390/ani12050628

**Published:** 2022-03-02

**Authors:** Vincent Nijman, Thais Q. Morcatty, Kim Feddema, Marco Campera, K. A. I. Nekaris

**Affiliations:** 1 Oxford Wildlife Trade Research Group, Oxford Brookes University, Oxford OX3 0BP, UK; tqueiroz-morcatty@brookes.ac.uk (T.Q.M.); mcampera@brookes.ac.uk (M.C.); 2 Business School, University of Western Australia, Perth, WA 6009, Australia; kim.feddema@research.uwa.edu.au

**Keywords:** Asian Songbird Crisis, CITES, conservation, Indonesia, illegal wildlife trade, social media

## Abstract

**Simple Summary:**

Throughout the world, wild-caught animals are traded in wildlife markets, but it is not always easy to disentangle what part of the trade is legal and what part is not. This may diminish the value of conducting wildlife market surveys. Conservationists narrowly focus on whether a species is legally protected, whereas in most countries there are several laws and regulations in place that guide the trade in wild-caught animals. Here we present empirical data from various species of birds recorded during wildlife market surveys in Indonesia and assess whether violations take place in terms of (1) protected species, (2) harvest quota, (3) welfare, (4) transport restrictions, and (5) importation. Our five distinctly different case studies showed that while it is challenging to distinguish legal and illegal aspects, in all cases it was evident that at least some aspects of the trade were in violation of Indonesia’s domestic legislation. By focusing on a wider range of legal restrictions, it is possible to get a good insight into the legality of wildlife trade, what interventions can be made, and overall, our study underscores the value of conducting wildlife trade surveys.

**Abstract:**

It is challenging to disentangle the legal and illegal aspects of wild-caught animals that are traded in wildlife markets or online, and this may diminish the value of conducting wildlife trade surveys. We present empirical studies on the trade in birds (ducks, owls, songbirds, non-passerines) in Indonesia (2005 to 2021). Based on visits to wildlife markets, wholesale traders, and monitoring of an Instagram account, we examine if five specific pieces of legislation (domestic and international) are adhered to: (1) protected species, (2) harvest quota, (3) welfare, (4) provincial transport restrictions, and (5) illegal import of CITES-listed species. Our five distinctly different case studies showed that in each case, certain rules and regulations were adhered to, whilst others were violated to varying degrees. When trade involved non-protected species, there was frequently a lack of harvest quotas or trade occurred above these allocated quotas. Basic welfare provisions were regularly and habitually violated. Visiting wildlife markets and recording first-hand what is openly offered for sale is a highly reliable, verifiable, and valuable method of data collection that can give insight in numerous aspects of the animal trade. Our research provides support for recognising the urgency for the government to take appropriate action to curb all the illegal aspects of the bird trade in Indonesia.

## 1. Introduction

The trade in birds in Indonesia is huge [1,2,3,4,5] and affects many species that are native to the country as well as those species that use the archipelago nation as a stopover on their north–south or south–north migration. Some of this trade is legal, some of it clearly is not [2,3], and there is an interconnectedness between the legal and illegal wildlife trade. Here we aim to disentangle the legal and illegal aspects of the bird trade in Indonesia by examining data from empirical studies. The ability to parse out the legal and illegal wildlife trade has implications for how legislation is enforced in an equitable and effective way. Additionally, recognizing the complexity of relationships between legal and illegal trade highlights the necessity for conservation organizations not to restrict their focus to illegal trade but to approach trade in a holistic manner. By doing so, we also demonstrate the value of conducting empirical studies on the wildlife trade through visits of wildlife markets, wholesale traders, and monitoring of online trade as it allows us to gain insight into both the magnitude and legality of the Indonesian bird trade. 

Bird-keeping is a highly popular pastime in Indonesia and in recent years there has been much focus on the trade in songbirds, and the effect this trade has on the conservation status of imperilled birdlife [1,4,5]. The passion for keeping songbirds is driven primarily by the aesthetic appreciation of song. Most consumers buy birds to be kept at home, but a small but important minority enter their songbirds into song contests [6,7]. In addition to the appreciation of song, birds may be valued for their physical appearance and colourful plumage [8], their ability for speech (e.g., parrots [9]), their links to popular culture (e.g., owls [10]), or their rarity and/or protected status (e.g., eagles [11]). In these cases, both songbirds and non-songbirds are traded. Apart from keeping them alive as pets, wild birds are traded within Indonesia for their meat [12]. Trade for other purposes, such as traditional medicine, affects far fewer species and individuals [13]. The main locations where birds, and indeed other wildlife, are offered for sale are wildlife, animal, or bird markets (in Indonesian these are known as *pasar satwa*, *pasar hewan*, or *pasar burung*) that can be found in most major cities throughout western Indonesia. We refer to these as wildlife markets. The wildlife markets range from a few shops to large multistorey complexes where hundreds of vendors offer birds, mammals and, to a lesser degree, reptiles for sale. The animals on offer include domesticated animals (e.g., canaries *Serinus canarius*, chickens *Gallus gallus*, rabbits *Oryctolagus cuniculus*) and captive-bred non-domesticated animals (e.g., Javan pied starlings *Gracupica jalla*, common palm civets *Paradoxurus hermaphroditus*) but mostly deal in wild-caught animals (e.g., white-eyes *Zosterops* spp.; long-tailed macaques *Macaca fascicularis*) [1,2,4,5,7,8,9,10,11]. 

In ‘Sold for a Song’, Nash [14] summarised that “A substantial portion of Indonesia’s domestic bird trade involves a large number of forest species which will not receive adequate food and care in captivity, and which usually do not survive very long. Thus, much of Indonesia’s domestic trade in native species is a wasteful ‘cut-flower’ industry, requiring a constant collection of short-lived commodities.” Many of the birds that are bought in the wildlife markets by consumers are alive, sing beautifully, and are colourful (some altered by dying or bleaching). Once brought home, they often quickly wither away. Importantly, as when buying a bouquet of flowers, the consumer is perfectly aware and accepting that these birds will have a short lifespan. From Nash’s study [13] and many others (e.g., [2,3,5,8,15,16,17,18]), it is also clear that conditions along the trade chain (capture, transportation, transit) and in the wildlife markets prior to the point of sale are far from optimal, resulting in high mortality rates. The impact on wild populations can be profound. 

Data from December 1986 from the Pramuka wildlife market in Jakarta suggest that annually some 1,800,000 wild-caught birds were sold in this market [19] (Chng et al. [2] recorded similarly high numbers in June 2015). Turnover for the 15 most traded species averaged 69% per week, ranging from a low 17% for pin-tailed parrot-finches *Erythrura prasina* to a high 98% for white-headed munias *Lonchura maja* [19]. In 1993 Nash [14] made a second attempt to estimate the volume of the bird trade in this same market, but he focused on non-CITES listed birds only. Based on a one-day survey, again in Pramuka wildlife market, and a generalised two-week turnover period for all species as suggested by vendors, he estimated an annual turnover of ~480,000 wild-caught non-CITES listed birds in Pramuka [14]. Extrapolating this to western Indonesia he estimated that at the time at least 1,300,000 wild-caught non-CITES listed birds were sold annually. Unfortunately, Nash was not able to provide estimates for individual species, but he indicated that around 30 species were traded both in large volumes and in most wildlife markets (>500 individuals observed in at least 19 of the 37 surveys). Despite an increase in the number of studies reporting on various aspects of the wild animal trade as studied empirically in the wildlife markets it is worth considering the value of these studies in gaining insights into the intricacies of this trade and to evaluate the legality of this trade systematically. 

### 1.1. The Value of Conducting Empirical Studies on Wildlife Trade

Recently conflicting reports questioned the value of surveying wildlife markets or wholesale bird traders when it comes to obtaining insights into the Indonesian bird trade [20,21,22]. This is not unique to Indonesia and part of this debate centres on the illegality of the trade and how that influences one’s ability to record what is present. The methods used to investigate wildlife trade also depend on the question we are trying to answer, the time and funding available for the study, the pre-existing information available and how open or clandestine the trade is. Each method of investigation has advantages and limitations, and every situation must be considered unique when deciding the appropriate methods to use [23]. Market surveys are one method of gaining insight, as are conducting interviews with traders and/or consumers [15,24], analysis of traders’ diaries and logbooks [21,22], or government seizure records, visiting farms or breeding facilities [3] and monitoring the online trade. Challenges with detection and accurately recording of especially the illegal component of the wildlife trade have been recognized [25,26]. These challenges may differ between products and species (legally protected turtles may be openly displayed but equally legally protected mammals may not), between cities, between countries and it may differ over time (what was tacitly allowed ten years ago may no longer be). As such, different methods of conducting wildlife trade research are perhaps best seen as complementary [23]. 

Busina et al. [20] surmised that the reliability of trade estimates derived from open wildlife market surveys were disputable, deceptive and of questionable credibility. Focusing on one species of songbird, the Sumatran laughingthrush *Garrulax bicolor* for sale in the city of Medan in North Sumatra, they found that making visits to wildlife markets and recording what is openly for sale did not lead to clear evidence about the volume, scope, and dynamics of the actual trade in wildlife. Specifically, they singled out three reasons why surveys based on direct counting of individuals that are openly offered for sale were methodologically flawed [20]:

1. Vendors might be aware of the illegality of their activities, and little is known about how much they actually reveal about the volume and scope of the trade, but they asserted that in general vendors were not willing to share information with investigators.
2. There is no agreement between vendor records of what they sell and direct observations in the wildlife market.
3. Only a small number and a small proportion of the Sumatran laughingthrush available for sale were displayed in wildlife markets. These numbers varied little over time, i.e., shops mostly had a few birds on display at a time making it difficult to assess how many were sold during subsequent visits, and this hampered any effort to extrapolate findings.

Many of these challenges encountered by Busina et al. [20] seem to be specific to the wildlife market they visited, the species they focused on, the way they conducted their research, and the experience and perhaps preconceptions of the survey team (see Discussion). Others, including us, have surveyed the same wildlife market [4,27,28], have studied the same species [27,29,30], and have used the same methods in data collection and analysis [31,32], and clearly did see the value in surveys based on direct counting of individuals that are openly offered for sale.

### 1.2. Examining the Adherence to Legislation and Trade Regulations

There are several types of protection that wild birds receive in Indonesia, and various regulations are in place concerning the transport of wild-caught birds within Indonesia. Consequently, people can commit different types of crimes when harvesting, transporting, selling, buying, and owning wild birds. We here consider five potential violations, related to (1) nationally protected species, (2) harvest quota, (3) welfare of wild birds in trade, (4) movement of live birds between any of the 34 provinces, and (5) records of the import of non-native CITES listed birds into Indonesia. While several researchers have focused on one, two, or even three of these potential violations, there is a clear preponderance to focus on either protected species legislation for native birds [2,3,4,7,8,9,10,14,15,28] or CITES listings for non-native birds or ones that are exported [5,7,9,28,30], and not on a broad range of violations (but see [24]). 

Species can be protected by being included on the country’s protected species list (UU Nomor P.20/MENLHK/SETJEN/KUM.1/6/2018). When protected, one cannot catch, transport, keep, sell, or buy any individual of this species. Some legally protected species can be sold, but only second-generation offspring (or above), when bred in government approved facilities and when accompanied by the correct paperwork [5].

As a rule, species that are not legally protected cannot legally be traded for commercial purposes, but can be used for subsistence, for personal use or to resolve human-wildlife conflict. For a non-protected species to be traded commercially, firstly a request for a harvest or capture quota needs to be made by the regional natural resource management agency (BKSDA) to the Ministry of Forestry. Only when this permission is granted, and a harvest quota has been allocated, can a non-protected species be traded (Decree of the Ministry of Forestry 447 of 2003). Due to the allocation of harvest or capture quotas, only a limited number of non-protected species can be legally traded in Indonesia. For example, in 2016 a harvest quota was available for only ~80 bird species for all of Indonesia. The median harvest quota was 140 birds per species for the entire year. Thus, given the sheer number of species available in the wildlife markets (two days of surveying three of Jakarta’s animal markets resulted in 206 species of wild bird being recorded, 184 of which were native to Indonesia [2]), many species in the wildlife markets should not have been allowed to be traded as no harvest quota had ever been allocated. For those non-protected species for which there is a harvest quota, these allowances are often (very) low compared to the actual number of individuals present in trade [33]. This suggests that the majority have been caught outside the allocated harvest quota. 

In the Penal Code of 1918, the maltreatment of animals is addressed in Article 302. This includes light maltreatment (if they deliberately cause pain or harm to an animal or cause injury to the health of an animal or withhold the necessary sustenance from an animal that wholly or partially belongs to them and is under their supervision), and severe maltreatment (if the above causes an illness longer than one week, mutilation, serious harm of another nature, or death of the animal). The maximum punishments are three and nine months imprisonment for light and severe maltreatment, respectively [34]. Despite this legislation, clearly the basic welfare provisions are regularly or habitually violated in the wildlife markets, and during shipment to the markets [35,36]. 

We are not aware of any trader having been charged or prosecuted for violation of Article 302. As noted by Regueira and Bernard [37] trade conditions are frequently degrading; animals are caged in overcrowded compartments, without water and food, and suffer stress, fights, mutilations, and death, and those animals that survive frequently suffer abuse with consequences such as death or a greatly reduced life span. 

Species that are sold outside the province in which they are caught can only be transported across provincial borders if accompanied by the appropriate paperwork (Article 42 of Law Number 8 of 1999). These cross-provincial transport documents can only be available for birds for which a harvest quota is available, or that have been captured within their allocated quotas (this includes both native species that are listed on CITES and ones that are not). Non-native species that are listed on Appendix I or II of the Convention on International Trade in Endangered Species of Wild Fauna and Flora (CITES) can only be imported into Indonesia with the appropriate permits. In the year following import both the importing country (i.e., Indonesia) and the exporting country (if Party to CITES) reports the transaction to the CITES Secretariat. These records are publicly available in the CITES trade database. 

In addition to the above, bird traders must be in possession of a business permit (*surat izin usaha perdagangan*) and those that sell from permanent shops need to have a place of business permit (*surat izin tempat usaha*). We were not able to assess whether the traders we surveyed possessed these licenses, but it is worth noting that Miller et al. [26] for the province of West Kalimantan, found that 63% of bird shop owners had no permits, 24% lacked one of the two permits and only 13% possessed both permits. From this they concluded that most bird shop owners were not paying any property or commodity tax. In 2015, the United Nations General Assembly adopted Resolution A/RES/69/314, which called upon member states to make illicit trafficking in protected species of wild fauna and flora involving criminal groups a serious crime, in accordance with their national legislation and article 2 (b) of the United Nations Convention against Transnational Organized Crime (UNTOC). Serious crime, as defined here, is an offence that is punishable by four years or more in prison. The links between wildlife trade and serious organised crime has received attention in recent years [11,23,25,26,37,38,39,40,41,42,43,44] Within Indonesia, it is well-known that illegal wildlife trade occurs: everybody, including authorities, know where to find it, the subject is covered by the media, and the public is largely aware of the negative impacts associated with it [3,4,7,8,10,12,13,14,15,16,17,18,19,20,21,22,27,41,45]. What is less well-resolved is whether the trade in birds in the wildlife markets in Indonesia constitutes serious organised crime.

Our aim here is two-fold. Firstly, we want to demonstrate the value of conducting empirical studies on the wildlife trade through visits of wildlife markets, wholesale traders, and monitoring of online trade to gain insight in both the magnitude and legality of this trade. Secondly, we wish to disentangle the legal and illegal aspects of the wildlife trade by examining data from these empirical studies. Thirdly, we discuss the issue of serious organised crime when it comes to the trade in birds in the wildlife markets.

## 2. Materials and Methods

We have been conducting research on the Indonesian bird trade since the early 1990s; initially this concerned only animal market visits, but later this was complemented by obtaining data from traders and exporters, data from rescue centres and zoos, and, over the last ten years, trade over the Internet [2,3,10,11,23,33]. Our research focuses on wild bird species, comprising both wild-caught birds and, to a lesser extent, captive-born or captive-bred birds. None of the species we refer to here are domesticated. We present data from five different case studies that, when combined, give a good overview of various aspects of the bird trade in western Indonesia, i.e., trade in wandering whistling ducks *Dendrocygna arcuata* in Indonesian Borneo, trade in owls in Java, Sumatra and Bali, trade in strawheaded bulbuls *Pycnonotus zeylandicus* in Java, trade in orange-spotted bulbuls *Pycnonotus bimaculatus* and yellow-fronted bulbuls *P. goiavier* in one animal market in west Java, and finally, a study of non-passerine birds on an Instagram account from a trader based in Java. Data were collected over different time periods, in different settings, and focused on a wide range of bird species, including ones that are legally protected, ones for which harvest quotas are in place and ones for which no harvest quotas were allocated, and species that do not occur in Indonesia and that had to have been imported. This broad approach allows us to present a narrative that extricates the legal from the illegal aspects of the trade, or at least, provides a more nuanced overview of the trade in wild birds in Indonesia. At the same time, it allows us to evaluate the use of monitoring wildlife markets for obtaining insights into the Indonesian bird trade. For a summary of the five different studies, their locations, and the survey intensities, see Table 1.

### 2.1. Trade in Wandering Whistling Ducks in Indonesian Borneo

This study builds on prior work by Nijman et al. [31] and Fredriksson et al. [32]. Wandering whistling ducks are not included on Indonesia’s protected species list. Most of the study was conducted between June 2005 to April 2007 on and around Lake Jempang, Lake Melintang, and Lake Semayang in East Kalimantan. We conducted 23 monthly surveys on the premises of four duck wholesale traders near Lake Jempang in the province of East Kalimantan, Indonesian Borneo, to visually count the number of ducks that were present in their facilities. Over the same period, June 2005 to April 2007, independently from the surveys, monthly verbal reports were available from the duck wholesalers about their trade [23]. This included information on the number of ducks the traders recalled selling that month and how many duck harvesters were active on the lakes [32]. 

Our wandering whistling duck study is comparable to the study conducted by Busina et al. [20] in that data were collected through visual inspection of the number of birds that were available for sale and through discussions with wholesale traders. It differs in the longer duration (23 months vs. nine months) and the number of birds that were observed (10,567 vs. 461). Finally, over the same period, monthly surveys were conducted on Lake Jempang, Lake Melintang, and Lake Semayang to count the number of wandering whistling ducks in the wild. 

On an ad hoc basis, we continued to collect data until 2010, but by that time the number of duck harvesters on the lakes had declined considerably, most likely in response to declining numbers of ducks making the trade no longer profitable. 

For analysis, we log-transformed the data and we used a Pearson’s correlation coefficient to explore how well these different methods (direct observations and information provided by traders) matched. 

### 2.2. Trade in Owls in Java, Sumatra and Bali

Owl trade work builds on that by Nijman and Nekaris [10], who assessed whether an increase in trade of owls as novelty pets followed the release of the Harry Potter books and films. Most owls in Indonesia are not protected, but no harvest quotas have been allocated to any of these species. For the period 1986–2020, we obtained data from seven full inventories on the islands of Java, Bali, and Sumatra [8,14,17,19,28,29,30] each recording between 20,500 and 403,783 birds for a total of 805,813 birds. We calculated the percentage of owls in each study. In addition, for the period April 2012 to February 2020, we visited nine wildlife markets (three in Jakarta, three in West Java, and one in Central Java, Yogyakarta, and Bali each) and counted all birds on display. Owls were traded openly in the wildlife markets so there was no need to resort to undercover techniques. We walked through markets slowly, recording owls by typing the species and their numbers using a mobile phone or by memorising numbers and writing them in a notebook directly on leaving the market. Individuals were identified to species level, when possible, but many scops owls (*Otus* spp.) were still chicks and could only be identified to the genus level. 

### 2.3. Trade in Strawheaded Bulbuls in Java

The assessment of trade in strawheaded bulbuls is a follow-up study of Bergin et al. [46], who reported on the trade in this species in 12 wildlife markets in 8 cities on Java and Kalimantan over a one-year period (July 2014 to June 2015), during which 71 birds in total were observed. Strawheaded bulbuls are listed as Critically Endangered on the IUCN Red List and are protected in Indonesia, but commercial breeders do breed the species [47]. Captive-bred strawheaded bulbuls often, but not always, have closed legrings, so their presence is strong evidence of the birds having been captive-bred. Birds without legrings can be captive-bred or wild-caught. Wild-caught birds are rare and are desired by certain hobbyists; given that this affects the price and desirability, traders often unsolicited indicate whether the strawheaded bulbuls they had for sale were wild-caught. 

We selected six wildlife markets that were both representative and that we had sampled consistently over a long period for strawheaded bulbuls at least once for each of the years between April 2015 and March 2020, i.e., Pramuka and Barito in Jakarta, Sukahaji in Bandung, Kerkhof in Garut and Cikurubuk in Tasikmalaya, all in the province of West Java and PASTY (Pasar Satwa dan Tanaman Hias Yogyakarta) in the special district of Yogyakarta in the central part of the island of Java. Methods were similar as described above for the owl surveys. 

For each market and for each year, we calculated the mean number of strawheaded bulbuls we observed during our surveys. We calculated the mean number of birds that were recorded in each market, averaging data from the six years. We also calculated the mean number of birds that were recorded in a wildlife market for any given year, averaging data from the six markets for each of the six years. 

### 2.4. Trade in Yellow-Fronted and Orange-Spotted Bulbuls in Kerkhof Wildlife Market

We report on the trade in these two closely related inexpensive bulbul species through intense surveying of the Kerkhof wildlife market in Garut, West Java. We, and our team, first visited Kerkhof wildlife market in early 2012 and have visited it regularly ever since. Kerkhof comprises some 20 shops in Garut’s city centre, opposite the old Dutch cemetery (hence the name, Kerkhof meaning cemetery in Dutch). The small number of shops, and hence small number of traders, and the many visits over an extended period, allowed us to build up a high level of rapport. This in turn gave us unusually detailed insight into the bird trade in this market. Enforcement actions in Kerkhof are all but absent (or at least over the ten years we visited the market we did not observe any, nor did any of the traders indicate to us that enforcement actions had been taken while we were not there).

Orange-spotted bulbuls are not protected and there are no harvest quotas for the species; likewise, yellow-fronted bulbuls are not protected, but a small annual harvest quota is present (see Results). We conducted 76 fortnightly surveys (August 2016 to October 2019) with either or both species being recorded during each survey. In addition to the fortnightly surveys, we conducted 24 weekly visits, and this allowed us to estimate turnover (percentage of birds that were sold or had died within a seven-day period) [48].

### 2.5. Trade in Non-Passerine Birds on Instagram

The Instagram work builds on that by Nijman et al. [49] who monitored pet shop traders on the social media platform. While much of the focus on the bird trade in Asia is on songbirds (passerines)—hence the term Asian Songbird Crisis [1]—there are 23 other Orders of birds present in Indonesia and many of them appear in trade. One trader based in Java who specialised in offering non-passerine birds from all over Indonesia and abroad was monitored for a year. We used a passive, manual approach in our online trade survey to record, filter, classify, and assess legal and illegal trade [34]. We assume that birds that are included on Indonesia’s protected species list cannot be sold (a number of protected songbird species can be sold provided they are second generation captive-bred, such as the strawheaded bulbul, but this is of less relevance here as the trader specializes in non-passerines). Non-native birds that are listed on the appendices of CITES and for which there are no recent records of their import for commercial purposes into Indonesia and that are offered for sale, most likely also represent illegal trade. We did not interact with the trader or his customers nor did we access any personal profile pages. We only collected information that was publicly displayed. Data (from traders and correspondence between the trader and potential customers) were anonymized after cross-checking for duplicates, and no information after the monitoring session can be attributed to one person. Photographs that were uploaded onto the Instagram accounts were collected on the day of data collection and stored on an encrypted drive (cf. [50]). 

For each month, we counted how many birds were advertised for sale. We excluded reposts, viz., the same individuals that had not been sold in the previous month(s) were offered for sale again, and we took a conservative approach when estimating numbers (i.e., when we observed four individuals of a particular species in one month and then the next month we observed three, we assumed that these three were part of the original four rather than assuming we observed seven individuals). Occasionally photographs of passerine birds were posted, such as strawheaded bulbuls, but it was unclear if these were indeed offered for sale or if these were pets owned by one of the traders. Given that the trader’s specialty was clearly in non-passerine birds, we excluded the small number of passerines.

We compared the numbers of CITES-listed non-native bird species with data reported in the CITES trade database for the period 2000–2020. We extracted numbers of live birds or live eggs as reported by the importing country (i.e., Indonesia) and the exporting countries. 

## 3. Results

### 3.1. Trade in Wandering Whistling Ducks in Indonesian Borneo

#### 3.1.1. Observations

The trade in wandering whistling ducks was exclusively for meat and all the birds were locally sourced on Lake Jempang, Lake Semayang, and Lake Melintang. The mean number of duck harvesters active on the lakes differed significantly between months (χ^2^ = 88.64, df = 11, *p* < 0.0001) and showed a strong correlation with the number of ducks that were observed on the lakes in the following month (Pearson’s R = 0.898, R^2^ = 0.806, *N* = 10, *p* = 0.0004). Local consumption of wandering whistling duck meat was limited, and the birds were transported in trucks to the city of Amuntai in the province of South Kalimantan, some 500 km overland. In 2005, 2006, and part of 2007, the birds were transported alive, but live transports of wild birds became more restricted due to the risk of the spread of avian influenza and the implementation of the National Strategic Plan for Avian Influenza Control in Indonesia. To circumvent this, from 2007 onwards many more ducks were slaughtered on the premises of the traders near Lake Jempang, with the carcasses transported in ice coolers to South Kalimantan. 

**Figure 1:**
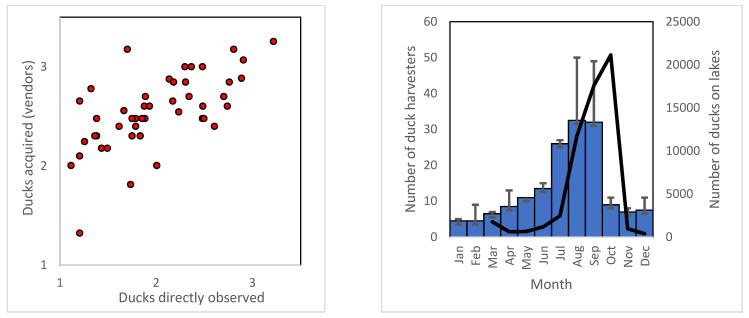
Illegal harvest and trade in wandering whistling duck *Dendrocygna arcuata* (June 2005 to April 2007). Left: Data from 23 monthly surveys of the premises of wholesale traders in East Kalimantan and from verbal reports of the number of ducks traded (both expressed on logarithmic scales) showing high levels of agreement. Right: Number of duck harvesters active in each month on Lake Jempang, Lake Semayang, and Lake Melintang (Mean + SEM) (bars) and the number of wandering whistling duck observed on these lakes during surveys (continuous line).

We observed 10,567 individuals at the premises of the four duck traders over the 23-month monitoring period. Recall data from these traders suggests that 26,629 wandering whistling ducks were traded. The monthly surveys of the premises and the verbal reports of the number of ducks acquired by these traders each month show that the site visits recorded two-fifths of that reported by traders (means of 179 ± 36 and 451 ± 49 ducks). There was strong correlation between the two methods (Pearson’s R = 0.457, R^2^ = 0.209, *N* = 23, *p* = 0.0003) (Figure 1). 

#### 3.1.2. Legislation, Regulation and Violations

The wandering whistling duck is not protected, and in 2005 to 2007 there was a zero-harvest quota for the species in place. In 2008, a quota of 500 birds was allocated for the province of East Kalimantan, 450 of these were intended for export, and all were to be traded alive. The quota was not renewed in 2009. Since 2018, a quota of 150 wandering whistling ducks is in place for East Kalimantan, for domestic use. Over the course of our studies, each year 10,000+ individuals were harvested and traded alive or as carcasses. This is well above the allocated quotas, which were only for the live bird trade, and in most years the cross-provincial transport of wild-caught birds was against avian influenza control rules.

Wandering whistling ducks were mostly captured with standing nets, stuffed in crates by the 100s in small boats, and transported to the premises of the traders near Lake Jempang. Here, they were placed in large holding facilities, again in large numbers, where they had access to water. For transport to South Kalimantan, they were placed in plastic crates normally used for broiler chickens, with no room to move and no access to food or water, and transported by road for 15 to 20 h. As such, basic welfare provisions were regularly and habitually violated.

### 3.2. Trade in Owls in Java, Sumatra and Bali

#### 3.2.1. Observations

**Figure 2:**
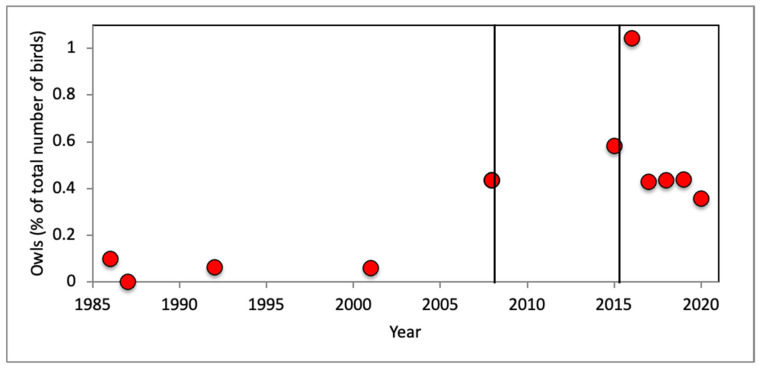
Trade in owls in Sumatra, Java and Bali over a 35-year period showing that the proportion of owls in trade increase markedly between 2001 and 2007. The vertical lines at September 2001 and January 2008 indicate the release the Indonesian translations of the first and the final installment of the Harry Potter series.

Owls have always been traded in small numbers in the wildlife markets of Sumatra, Java and Bali, but a clear increase in relative numbers have occurred post 2007. Prior to that, of the 380,914 birds that were observed and identified in these markets, up to 0.1% were owls. In more recent years, of the 424,899 birds observed and identified in these markets between 0.4 and 0.6% were owls (Figure 2). 

Between April 2012 and February 2020, we observed a total of almost 3000 owls of ten species in the nine wildlife markets on Java and Bali (Table 2). Mean numbers were highest in the Jatinegara wildlife market in Jakarta (44 owls per visit) and the PASTY wildlife market in Yogyakarta (19 owls per visit). By far the most common species were the scops owls comprising three-quarters of the total, but Australasian barn owl *Tyto javanica* and the Oriental bay owl *Phodilus badius* were also common in trade. BirdLife [51] indicated that the Oriental bay owl was extirpated from Bali. We observed four of them in trade in the Satria wildlife market, on three different occasions, and in addition, we observed two in the Beringkit wildlife market in Mengwi, also in Bali. This may suggest that the species is still present on the island, or, alternatively, that it is imported from Java, Sumatra or Borneo. 

While a large proportion of the owls in trade were chicks, nestlings, or fledgelings, these were all taken from the wild rather than being the result of commercial captive breeding operations. Traders indicated that most of the owls that arrive at the wildlife markets were taken from their nest. We did not observe any owls with closed legrings, and their general skittish nature indeed suggests that the owls we observed in the wildlife markets were taken from the wild. There are some commercial breeding operations for Australasian barn owls on Java [10] but these are to supply forest plantation owners.

#### 3.2.2. Legislation, Regulation and Violations

Between 2012 and 2020 when we conducted our surveys, one company PT Astra Agro Lestari, was given permission to capture up to 500 Australasian barn owls each year in the Sumatran province of Riau. In 1984 PT Astra Agro Lestari established its first palm oil plantation in Riau, and as of 2020 it manages almost 3000 km^2^ of plantations in western Indonesia. By 2019, it had distributed 6210 owl nest boxes throughout its plantations to reduce rat populations [52]. There are no harvest quotas for any of the other species of owls. As such, the trade is in violation of this when it comes to the trade in owls for pets in Java and Bali. 

Oriental bay owls, buffy fish owls *Bubo ketupa*, or eagle owls *Strix* spp. are largely confined to forested habitats, and they are most likely completely absent from the (deforested) Jakarta capital district. Given that almost a hundred of them were observed in the Jakartan wildlife markets, these must have been brought in from other provinces in Java (Banten, West Java) or from Sumatra or Borneo. In the absence of a harvest quota, the cross-provincial transport of these live birds would not have been permitted. While owls were generally well taken care of when fully-grown, a large number of owls, and especially a large number of scops owls, were nestlings or fledglings and must have been taken from their nests. These nocturnal species were often displayed in small carboard boxes, fully exposed in the hot sun, without access to proper food and parental care. 

We expect mortalities to have been high. The absence of the most basic welfare provisions for these birds is in violation of the Penal Code of 1918.

### 3.3. Trade in Strawheaded Bulbuls in Java

#### 3.3.1. Observations

We observed a total of 476 strawheaded bulbuls (Table 3). The numbers differed between the six wildlife markets, with mean numbers for PASTY wildlife market being ten times of more than the means for Barito, Kerkhof, or Cikurubuk Wildlife markets. These differences were not present when we compared it over the six years (2015 to 2020).

#### 3.3.2. Legislation, Regulation and Violations

While the strawheaded bulbul is listed as a protected species, trade in captive-bred individuals is permitted. While only a proportion of the birds we observed in the markets had closed legrings, evidencing that the birds were captive-bred, we consider it likely that some of the birds without legrings were also bred in captivity [46]. Discussions with traders in Jakarta, Bandung, and Yogyakarta revealed that most birds were referred to as Sumatran strawheaded bulbuls and, more rarely, Indonesian Bornean strawheaded bulbuls, but this included captive-bred birds (e.g., a strawheaded bulbul bred in captivity in Java would still be referred to as a Sumatran strawheaded bulbuls if that is where the stock originated from). Although it is unclear what proportion of the supply of the strawheaded bulbuls are sourced from the wild, at least some traders indicated that wild-caught individuals were considered superior because of their higher song quality. There was therefore an incentive to stock wild-caught birds over captive-bred individuals if traders could acquire them.

With nearly 500 of these Critically Endangered birds observed in trade in the six markets combined, it is equally correct to conclude that at least some were sourced from the wild; these birds caught have been caught in Sumatra, in Indonesian Borneo, in West Malaysia, and less likely, from Java. Given that the species is protected the movement of them between provinces would not have been permitted. While import from West Malaysia would require CITES permits, Indonesia has not reported the import of even a single strawheaded bulbul going back to the year 2000. Overall, we found limited evidence of illegal trade, but not all rules and regulations with regards to properly documenting that captive-bred origin of the individuals offered for sale were adhered to. For instance, we rarely observed the presence of proper documentation of the captive-bred status for the birds with legring (and never for the ones without). While the birds were mostly caged individually, given the high prices paid for strawheaded bulbuls, the birds were generally well taken care of, and few explicit violations of animal welfare regulations were observed. 

### 3.4. Trade in Yellow-Fronted and Orange-Spotted Bulbuls in Kerkhof Bird Market

#### 3.4.1. Observations

**Figure 3:**
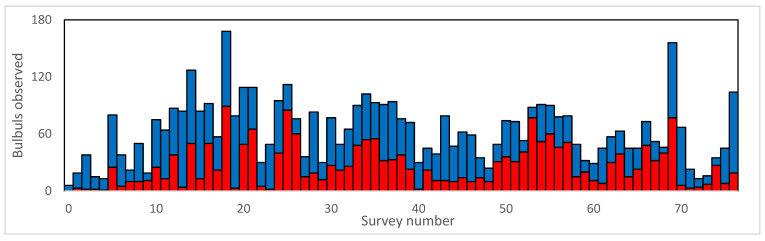
Numbers of orange-spotted bulbul *Pycnonotus bimaculatus* (red bars) and yellow-fronted bulbuls *P. goiavier* (blue bars) recorded openly offered for sale in 76 fortnightly surveys (August 2016 to October 2019) at Kerkhof wildlife market in Garut, West Java, showing substantial variations in availability.

We observed 2047 orange-spotted bulbuls and 2798 yellow-fronted bulbuls in Kerkhof wildlife market in Garut, West Java. The number of orange-spotted bulbuls and yellow-vented bulbuls recorded during 76 fortnightly visits shows large fluctuations in numbers offered for sale (means 27 and 36, range of 0 to 89 and 6 to 85 birds for orange-spotted bulbuls and yellow-vented bulbuls, respectively), and there was no significant correlation between the volumes of both species in trade (Pearson’s R = 0.175, *p* = 0.129) (Figure 3). This suggests that rather than vendors keeping approximately the same number of openly displayed individuals at all times regardless of the actual number of birds they have sold or could have in stock (as suggested by [20]), there was not only a large variability in the numbers on display, but this also differed between these two similar species. Kerkhof is a small local market and unlike some of the larger markets (Jakarta, Medan, Surabaya) does not cater for the wholesale trade and the numbers reflect both local supply and local demand. 

For both species, on 13 of the 24 weekly revisits the number of birds had declined. From this we estimate that the minimum turnover for orange-spotted bulbuls was 52.2 ± 7.3% week^−1^ and for yellow-vented bulbuls it was 38.2 ± 5.9% week^−1^. With a mean of 27 and 36 birds present this translates to the sale (or death) of 733 ± 102 orange-spotted bulbuls and 715 ± 110 yellow-vented bulbuls each year.

#### 3.4.2. Legislation, Regulation and Violations

For yellow-fronted bulbuls there is a harvest quota in place whereby each year 50 to 200 birds can be harvested from the province of Bengkulu on the island of Sumatra and 200 and 250 in the provinces of Central and South Kalimantan. The low value of the bird makes it economically not viable to transport any of these birds for hundreds of kilometres to a small market in western Java. Furthermore, the numbers we observed first-hand in the Kerkhof wildlife market exceeds the annual quota for all of Indonesia for the four-year study period. Likewise, our estimates of the number of yellow-vented bulbuls sold in Kerkhof wildlife markets equals or exceeds most year’s quota for all of Indonesia. In the absence of harvest quotas for orange-spotted bulbuls all trade is illegal. 

Of all the trade we observed in the animal markets in Sumatra, Java, and Bali, the trade in “cheap songsters” such as orange-spotted and yellow-fronted bulbuls is the one that adheres least to any animal welfare standard or regulation. When the bulbuls arrive in the markets, they are tightly packed in transport cages with several dozen or even over a hundred birds together. These then are habitually displayed in cramped in cages with dozens of birds packed together, and these are often exposed to the heat and direct sun. More often than not, one or more dead birds are found on the bottom of the cage, and these may be joined by one or more near-morbid ones.

### 3.5. Trade in Non-Passerine Birds in Instagram

#### 3.5.1. Observations

The one trader in Java, over the course of 12 months, we conservatively estimate that they offered 281 individuals of 36 species for sale on their Instagram account (Table 4). Parrots were especially abundant, both in terms of number of individuals and number of species, followed by birds of paradise and, to a lesser degree, galliforms. Only seven species are found on Java, and the others were imported mainly from eastern Indonesia (Papua, Lesser Sunda Islands, and the Moluccas) or, in smaller numbers, western Indonesia (Sumatra, Borneo) or Sulawesi. Three species of parrot were not native to Indonesia and must have been imported. 

#### 3.5.2. Legislation, Regulation and Violations

For only one of the ten species that were not included on Indonesia’s protected species list, the wandering whistling duck, was there a harvest quota for 2020. A total of 150 birds were allowed to be harvested in East Kalimantan and 25 in the province of Papua. It is possible that the two birds that were offered for sale in October were part of this quota, but it is equally likely that the birds were sourced more locally from Java. 

For the red-fronted parrot and the blue-eyed cockatoo, not a single individual was reported as imported since 2000, and Indonesia reported the import of four African grey parrots *Psittacus erithacus* (two from Singapore, one from Canada, and one from France) all for personal effect. 

These are most likely someone’s pet and were not intended for commercial trade. Exporting countries, however, did report the export to Indonesia. For the African grey parrot, exporting countries report the export of 5962 African grey parrots to Indonesia, the majority coming from South Africa (4993) and Bahrein (320). 

For the blue-eyed cockatoo *Cacatua ophthalmica* Singapore reported the export of two individuals in 2012 for personal effect (hence not for commercial trade) and in 2016 South Africa reported the export of 30 individuals, for commercial trade purposes. For the red-fronted parrot *Poicephalus gulielmi* exporters reported the import of 296 individuals over the twenty years period, mostly from Mali (130 individuals), Congo DRC (110), and South Africa (52), all for commercial purposes.

Some birds were caged individually, others in pairs or in small groups. Given the high prices paid for the birds, and as far this is possible to access from photos and videos alone, they appeared to be well taken care of and few clear violations of animal welfare regulations were observed. 

## 4. Discussion

### 4.1. Disentangling Legal and Illegal Wildlife Trade

In recent years it has become clear that Indonesia is the centre of the Asian Songbird Crisis [1,5,7,8,15,26,28,53,54,55,56,57] including a trade in a substantial number of species that are legally protected [58]. Our aim was twofold: to disentangle the legal and illegal aspects of the wildlife trade by examining data from empirical studies, and to evaluate whether visits of wildlife markets, wholesale traders, and monitoring of online trade would give us insights in both the magnitude and legality of this trade. A first step in trying to curb the illegal aspect of the wildlife trade, and to better regulated the legal trade, is to obtain an accurate picture of the number of species that are traded, their numbers, where the trade occurs, and how trade networks operate. This should be seen not as a one-off process but given the dynamic aspects of at least some parts of the wildlife trade, something that should be done continuously or at least regularly. While much of what Nash [14] wrote in the early 1990s or what Shepherd [8] wrote in the late 2000s with regards to the trade in wild-caught birds still rings true to this day, important aspects—including what species can be traded, species composition, regulation—has changed. Contemporary, high-quality data on all aspects of the wildlife trade continues to have value in setting the conservation agenda.

Our five distinctly different case studies showed that while it is challenging to distinguish legal and illegal aspects, in all cases it was evident that at least some aspects of the trade were in violation of Indonesia’s domestic legislation. In two of the case studies, there were clear violations of Indonesia’s protected species legislation. In three cases, there were violations of the harvest and trade quota. In three cases, it was evident that the welfare of the animals in trade was severely compromised resulting in violation of Indonesia’s Penal Code. In four cases there was no evidence to suggest that vendors adhered to regulations dealing with interprovincial transport of life animals. Finally, in two cases there was evidence to suggest that CITES-listed bird species, non-native to Indonesia, must have been imported into the country without import permits having been issued (Table 5).

Gönner et al. [58] regularly recorded over a thousand wandering whistling ducks on Lake Jempang, Lake Semayang, and Lake Melintang between 1997 and 2004, and this agrees with our observations on these same lakes. Very few were recorded from 2006 onwards [58], quite likely due to overharvesting. The large-scale trade in wandering whistling ducks from East to South Kalimantan came to a halt probably after 2010 not because of a diminishing demand or because of enforcement of existing legislation, but because of economic reasons [59,60]. It clearly represented a boom-and-bust trade, and once large-scale trapping had brought down the numbers of ducks considerably, it was no longer profitable to continue with this trade. 

### 4.2. The Value of Conducting Market Surveys

While certainly not perfect, visiting wildlife markets and recording first-hand what is openly offered for sale is a highly reliable, verifiable and valuable method of data collection that can give insight in numerous aspects of the animal trade. Even in cases where part of this trade happens in back alleys or out of sight, documenting what is openly on offer is a first step in gaining a better understanding into this trade. While it is tempting for researchers, conservationists, and NGOs to focus on the (illegal) trade in a small number of high-profile species, often the same conservation outcomes (curbing illegal trade, improving regulations, enforcing existing legislation, etc.) can be achieved by focusing on those species that are covered by the same legislation as the high-profile species but where it is easier to obtain a complete picture of the trade. Thus, rather than focusing on just orangutans *Pongo* spp., a collaborative project between the Indonesian MoF and TRAFFIC targeted orangutans and gibbons [61]. The latter were traded alongside the orangutans, in greater numbers, with middlemen, traders, and exporters more willing to divulge any details, and thus ultimately providing better insight into the illegal trade in all apes [49]. 

We show the breadth and depth of insights gained from conducting surveys in wildlife markets and wholesale bird traders to on both the legal and illegal aspects of the bird trade. While Busina et al. [20] rightly raise concerns about illegality of the songbird trade in Indonesia and how this affects surveying, it is important to stress that the wildlife markets in Indonesia are open to the public, are societally accepted, are officiated and visited by presidents and governors, and offer a wide range of products and services (animals, animal supplies and accessories, food, and drinks for humans, etc.). Legally protected species, while invariably present, make up a small proportion of what is on offer. When taking domesticated birds into account these numbers drop significantly. Many of the wildlife markets are situated in the same location, and shops have been run by the same families for decades. Certainly, when it comes to the trade in birds, vendors in wildlife markets have very little to fear in terms of being fined, having their birds confiscated or of being prosecuted for violation of protected species, harvest, or animal welfare laws [38]. The scale at which the trade takes place, i.e., 10,000 birds traded for meat clear for all to see [12,32,50,62] is evidence that the authorities either turn a blind eye or are colluding with traders. It was not uncommon for us to see law enforcement officers passing through the wildlife markets past numerous legally protected species without taking any apparent action. 

Busina et al. [20] noted that vendors might be aware of the illegality of their activities, that very little is known about how much they actually reveal about the volume and scope of the trade, and that in general vendors are not willing to share information with surveyors. We have always been pleasantly surprised about the amount of information and detail traders are willing to share, even where this involves trade in legally protected species, and similar conclusions were arrived at by a series of studies on the bird trade in West Kalimantan [15,24]. We did not find any noticeable differences when the work was done by local Javanese or Sundanese researchers, mixed teams of foreign and Indonesian researchers or foreign researchers only [63,64]. We are convinced that vendors are aware of the illegality of their activities when it comes to them offering protected species for sale, but in the near-complete absence of enforcement this does not alter how they try to sell these animals. It is the vendor’s their business to know about regulations and legislation as it affects price, availability, and demand. Consequently, it is difficult to see how this trade can continue without the explicit knowledge and involvement of the authorities. 

It is true that visits to wildlife markets, even when regularly revisited over long periods, do not give a picture of the trade in a species in its entirety, as there are always additional wildlife markets that have not been visited, there is online trade whereby birds are advertised on Facebook and Instagram, there are commercial traders and breeders that supply directly to customers and there are indeed wholesale dealers who supply a domestic and international clientele. However, wildlife market surveys do give insight into what is traded then and there and as many of the wildlife markets, in Southeast Asia at least, have been operational for decades they allow researchers to monitor changes over time [2,4,8,17,19,27,28]. In response to certain external events, such as outbreaks of avian influenza, COVID-SARS, Ebola, and COVID-19, wildlife markets may close temporarily, but hitherto these closures are rarely permanent and trade returns to (near) normal levels [65,66,67,68]. Whether these temporary closures also lead to a change in species composition—with different species being offered for sale before and after the closure—is a topic that deserves further study. 

We argue that first-hand observations of birds in wildlife markets, certainly when done over extended periods of time, gives a much more reliable record of what is offered for sale, where and when, than relying on second- or third-hand information, based on what someone, with unknown intentions, recorded without having a means of verifying this [20]. In addition, as shown here, first-hand observations allow for the assessment of how well vendors adhere to welfare standards. This is increasingly recognised as an important component to assess sustainability of harvest and trade [64,69,70,71]. Baker et al. [70] concluded that with regards to welfare and wildlife trade, a reasonable aspiration was to eradicate (or limit) that part of the trade that on welfare grounds is deemed irretrievably unacceptable and to improve animal welfare for the remaining parts. Welfare, furthermore, can also be a tool for law enforcement, as attention to the welfare needs of trafficked wildlife can expose the modus operandi and become a tool for law enforcement [71]. Specifically, the relative high welfare needs of wild-caught birds (relative to for instance plants, reptiles, and certain mammals) necessitate a fast movement through the trade chain and this requires both an organised infrastructure and a good degree of planning. Authorities wishing to respond to the illegal trade can use these specifics in planning and executing their actions. 

### 4.3. Legality of the Trade and Organised Crime

Miller et al. [24] noted that in West Kalimantan more than half of wildlife markets could be closed or at least sanctioned using regulations that are unrelated to the trade or exploitation of natural resources (because of lack of business permits or issues related to taxation). The noted that in the absence of the correct permits to run or own any sort of business, the songbird shop owners were not paying any sort of property or commodity tax [24]. If this finding holds true across Indonesia, including Java and Bali where we conducted most of our surveys, there may be an opportunity to leverage non-wildlife regulatory laws to reduce the volume of the songbird trade [24]. Our findings support the claim that a multifaceted approach to wildlife trade leveraging a variety of legal, economic, and demand reduction strategies is needed. 

As argued by Regueira and Bernard [44], wildlife markets (in their case Brazilian, for us Indonesian) are real biodiversity sinks and both society and authorities are underestimating the mid and long-term damage these markets are causing. Properly measuring the number of animals involved still is a difficult and challenging task. Conservation science can contribute by producing and refining the necessary data to assess the real impact of illegal wildlife trade [c.f., [72,73]]. However, research alone will not be able to fight it. Recognising the reality of trade, we and others have observed in the wildlife markets in Indonesia will be part of that—this is not a one-off realisation but it is something that needs to be made clear to all stakeholder involved, and this needs to be repeated, over and over. Illegal wildlife trade must be faced as an environmental, economic, and social problem. Some may argue that we can consider the wildlife trade in Indonesia as presented here as a “disorganized criminal network” that, compared to organized criminal networks, are less structured, not monopolistic, not territorial, and do not employ violence as a tool [74]. To label the traders and associates as organized criminal groups is also correct as for the majority of the trade we observed, those involved comprised a structured group of at least three people (that did not meet randomly), and this group existed for a period of time and acted in concert with the aim of committing one or more serious crimes or offences, in order to obtain, directly or indirectly, a financial or other material benefit [75].

We argue that following to the definitions of the UNTOC a significant part of the trade in birds in Indonesia should be considered a serious crime (as it is punishable by four years or more in prison) and that there is an urgent need to take appropriate actions to curb this trade. However, as we have shown, while it may be challenging to disentangle the legal from the illegal aspects of the bird trade in Indonesia, a wide range of offences with regards to wildlife protection and regulation are committed in the wildlife markets on a regular basis. There is no need to focus ‘just’ on protected species legislation, as there are many other laws, rules, and regulations that are habitually violated by wildlife traders. These include, but are not restricted to, violation of animal welfare legislation, interprovincial transport of animals without permits, sale of non-protected species above agreed quotas, sale of non-native CITES listed species without there being evidence that these species were ever legally imported, failure to register as a business operation, and an absence of place of business permits. Linked to this, and perhaps most easy to follow up on, is that many wildlife traders appear not pay to any property or commodity tax.

Similar to efforts to reduce poaching [76] creating a greater awareness of this, and incentivizing traders to abstain from offering protected wildlife for sale as well as potential consumers from buying this, could be more effective than focusing on law enforcement only.

## Figures and Tables

**Table 1:** Summary of the studies. Monthly surveys are defined as visits to a specific market that are temporarily separated by at least one month; surveys in Garut were fortnightly.

Species	Purpose	Monthly Surveys (Period)	Location	Individuals Observed (Species)
1. Wandering whistling duck	Meat	23; 2005–2010	Jempang, East Kalimantan	10,567 (1)
2. Owls	Novelty pets	166; 2012–2020	Sumatra, Java, Bali	3002 (10)
3. Strawheaded bulbul	Song, competition	142; 2015–2020	Western and central Java	476 (1)
4. Bulbuls	Song	76; 2016–2019	Garut, West Java	4845 (2)
5. Non-passerine birds	Novelty pets	12; 2020	Instagram (Java)	281 (36)

**Table 2:** Trade in owls on Java and Bali (2012–2020) showing mean numbers per survey for the most traded species and totals for each wildlife market and species. Species: Australasian barn owl *Tyto javanica*; Oriental bay owl *Phodilus badius*; buffy fish owl *Bubo ketupa*; Sunda scops owls *Otus lempij*.

Province Wildlife Market (Surveys)	*T. javanica*	*P. badius*	*B. ketupa*	*O. lempij*	*Otus* spp.	Other Owls	Total
DKI Jakarta							
Barito (25)	0.56	0.40	0.04	3.08	7.40	1.28	319
Jatinegara (29)	7.03	2.03	0.28	19.55	13.69	1.90	1290
Pramuka (13)	0.77	0.46	0.08	7.62	7.00	0.38	212
West Java							
Sukahaji, Bandung (24)	2.13	0.33	0.13	3.17	5.13	3.25	339
Kerkhof, Garut (28)	0.29	0.57	0.07	4.89	0.57	0.11	182
Plered, Cirebon (10)	1.90	0.40	0	2.80	2.50	0.10	77
Central Java							
Karimata, Semarang (16)	2.13	0.44	0	4.19	0.94	0	123
DI Yogyakarta							
PASTY, Yogyakarta (11)	4.00	1.00	0.09	12.00	1.64	0.18	208
Bali							
Satria, Denpasar (10)	0.70	0.40	0.30	3.20	3.60	0.40	86
Total	391	125	19	1,215	906	180	2836

**Table 3:** Number (mean ± SEM) of strawheaded bulbuls *Pycnonotus zeylandicus* observed in six wildlife markets in Java, Indonesia. The mean number of birds observed by year is calculated by giving equal weight to each wildlife market.

Bird Market (Surveys)	Mean ± SEM	Year (Surveys)	Mean ± SEM
Pramuka, Jakarta (18)	9.86 ± 1.79	2015 (11)	4.31 ± 2.63
Barito, Jakarta (18)	1.03 ± 0.52	2016 (19)	5.07 ± 2.25
Sukahaji, Bandung (28)	2.37 ± 0.65	2017 (45)	6.34 ± 3.89
Kerkhof, Garut (42)	0.44 ± 0.23	2018 (34)	5.19 ± 2.41
Cikirubuk, Tasikmalaya (26)	0.88 ± 0.50	2019 (24)	3.38 ± 2.05
PASTY, Yogyakarta (10)	15.46 ± 1.93	2020 (9)	5.75 ± 2.90

**Table 4:** One year of advertisements on Instagram from one pet shop based in Java, Indonesia. All species occur in Indonesia, apart from the three indicated in bold. Protected refers to the species being included on Indonesia’s protected species list; Quota refers to the harvest quota for 2020 for non-protected species; Occurs on Java refers to whether the species is native to the main island of Java (excluding vagrants).

Species	Protected	Quota	Occurs on Java	Birds Recorded (Months)
Cassowary *Casuarius* spp.	Yes			17 (4)
Wandering whistling duck *Dendrocygna arcuata*		175	Yes	2 (1)
Crested partridge *Rollulus rouloul*		0		19 (2)
Green peafowl *Pavo muticus*	Yes		Yes	23 (4)
Sumatran peacock-pheasant *Polyplectron chalcurum*	Yes			2 (1)
Victoria crowned-pigeon *Goura victoria*		0		9 (3)
Rose-crowned fruit-dove *Ptilinopus regina*		0		7 (1)
Stilt *Himantopus* spp.	Yes		Yes	26 (2)
Javan hawk-eagle *Nisaetus bartelsi*	Yes		Yes	1 (1)
Bonelli’s eagle *Aquila fasciata*	Yes			1 (1)
Eurasian hoopoe *Upupa epops*		0	Yes	4 (1)
Rhinoceros hornbill *Buceros rhinoceros*	Yes		Yes	3 (2)
Wreathed hornbill *Rhyticeros undulatus*	Yes		Yes	2 (1)
Sulawesi wrinkled hornbill *Rhabdotorrhinus exarhatus*	Yes			2 (1)
Palm cockatoo *Probosciger aterrimus*	Yes			21 (8)
Yellow-crested cockatoo *Cacatua sulphurea*	Yes			16 (7)
Moluccan cockatoo *Cacatua moluccensis*	Yes			12 (8)
Sulphur-crested cockatoo *Cacatua galerita*	Yes			3 (1)
White cockatoo *Cacatua alba*	Yes			36 (7)
**Blue-eyed cockatoo *Cacatua ophthalmica***				1 (1)
Vulturine parrot *Psittrichas fulgidus*	Yes			17 (6)
Moluccan king parrot *Alisterus amboinensis*	Yes			2 (2)
Eclectus parrot *Eclectus roratus*	Yes			35 (7)
Blue-backed parrot *Tanygnathus sumatranus*		0		4 (1)
Large fig-parrot *Psittaculirostris desmarestii*		0		1 (1)
Black lory *Chalcopsitta atra*		0		2 (2)
Red-and-blue lory *Eos histrio*		0		1 (1)
**African grey parrot *Psittacus erithacus***				5 (2)
**Red-fronted parrot *Poicephalus gulielmi***				2 (1)
Flame bowerbird *Sericulus ardens*	Yes			4 (2)
Lesser bird-of-paradise *Paradisaea minor*	Yes			3 (3)
Red bird-of-paradise *Paradisaea rubra*	Yes			3 (2)
Riflebird *Ptiloris* spp.		0		4 (2)
Wilson’s bird-of-paradise *Cicinnurus respublica*	Yes			3 (2)
King-of-Saxony bird-of-paradise *Pteridophora alberti*	Yes			2 (1)
12-wired bird-of-paradise *Seleucidis melanoleucus*	Yes			4 (2)

**Table 5:** Disentangling the legal and illegal aspects of the trade in birds in Indonesia. Listed are violations, presumed or evidenced, of protected species legislation, capture above permitted harvest quotas or capture in the absence of harvest quotas, violation of animal welfare legislation, violations of provincial transport restrictions, and the import of CITES listed birds in the absence of import permits. See text for details.

Species	Protected	Quota	Welfare	Transport	Import
1. Wandering whistling duck	No	Yes	Yes	Yes	No
2. Owls	No	Yes	Yes	Yes	No
3. Strawheaded bulbul	Yes	No	Not obvious	Yes	Yes
4. Bulbuls	No	No	Yes	No	No
5. Non-passerine birds	Yes	Yes	Not obvious	Yes	Yes

## Data Availability

The data presented in this study that are not yet included in the paper are available on request from the corresponding authors.
